# A New Preservative Process

**Published:** 1889-11

**Authors:** 


### A NEW PRESERVATIVE PROCESS.

It is a well-known fact that the oxygen of the air is the most essential element for the support and maintenance of life, whether vegetable or animal, and when this element has been exhausted or removed from any medium or enclosure life of every kind becomes extinct. It is also a well established fact that decomposition or fermentation is due to and carried on by the growth and development of the lower forms of life, which organisms are capable of extracting from the different fluids in which they multiply sufficient oxygen for their support. If, therefore, the oxygen contained in the various liquids to be preserved is entirely eliminated by some efficient mechanical process and then a pure antiseptic gas introduced to take its place, the fluids prepared in this manner will keep perfectly sweet and preserve their normal condition.

This is precisely what is accomplished by the process of the company, and it is in this condition that cider, wines, beer, ales and milk can be prepared and kept indefinitely without any regard to outside conditions of the atmosphere. One of the practical and valuable features of the process is that all the liquids named can be put up in siphons and supplied in this most convenient form to the consumer. The latter can then use any desired quantity of the contents of the siphon and keep the remainder intact for future use. For invalids and those who do not care to use alcoholic liquors the process permits the pure expressed juice of the fruit being preserved with its natural flavor and bouquet. The value of pure carbonated milk for ready consumption will also be appreciated by physicians. Just as mineral waters are delivered to the consumer by the bottler or grocer, so can siphons of assorted liquors, such as beer and ale, be delivered in a similar manner. The fact that the drawing of corks and consequent exposure of the liquid to deterioration can be dispensed makes the new process economical, practical and convenient

---

Deep Breathing.—Deep inhalation is said to be the key to health and beauty. Breathing, like learning, is a dangerous thing taken in small draughts. Breathe, as well as drink, deep if you would be refreshed. Men of science frequently assert that if we breathed properly, we would have no impure blood.

How is this deep breathing done? by what process? "Simply thus. Stand, inhale deeply, fully, completely. As you do so, let the waist expand, and don't be afraid to have the abdomen protrude. At the last of the inhalation let (don't *make*) the chest expand. Let the air out gradually, and repeat the operation five or ten times. He who thinks he must begin inhalation by *making* the chest spread, falls into a serious error, because this course *prevents* complete inhalation. Thirty or forty deep inhalations every morning in as pure air as possible, will do more to keep the circulation of the blood good, the blood itself pure, the lungs well and strong, and the movement of the secretions active, than all the medicine any one can take."

Ladies who incase themselves in corsets which narrow their waists to painful proportions, or no proportions at all, cannot practice deep inhalation. Neither is it for the tailor-made girl ; she has all she can do to breathe at all, and stagger under the weight of heavy skirts which hang upon her bustle.

